# A SUPER Powerful Method for Genome Wide Association Study

**DOI:** 10.1371/journal.pone.0107684

**Published:** 2014-09-23

**Authors:** Qishan Wang, Feng Tian, Yuchun Pan, Edward S. Buckler, Zhiwu Zhang

**Affiliations:** 1 School of Agriculture and Biology, Shanghai Jiaotong University, Shanghai, China; 2 National Maize Improvement Center of China, China Agricultural University, Beijing, China; 3 United States Department of Agriculture (USDA) – Agricultural Research Service (ARS), Ithaca, New York, United States of America; 4 Institute for Genomic Diversity, Cornell University, Ithaca, New York, United States of America; 5 Department of Animal Science, Northeast Agricultural University, Harbin, China; 6 Department of Crop and Soil Science, Washington State University, Pullman, Washington, United States of America; University of North Carolina, United States of America

## Abstract

Genome-Wide Association Studies shed light on the identification of genes underlying human diseases and agriculturally important traits. This potential has been shadowed by false positive findings. The Mixed Linear Model (MLM) method is flexible enough to simultaneously incorporate population structure and cryptic relationships to reduce false positives. However, its intensive computational burden is prohibitive in practice, especially for large samples. The newly developed algorithm, FaST-LMM, solved the computational problem, but requires that the number of SNPs be less than the number of individuals to derive a rank-reduced relationship. This restriction potentially leads to less statistical power when compared to using all SNPs. We developed a method to extract a small subset of SNPs and use them in FaST-LMM. This method not only retains the computational advantage of FaST-LMM, but also remarkably increases statistical power even when compared to using the entire set of SNPs. We named the method SUPER (Settlement of MLM Under Progressively Exclusive Relationship) and made it available within an implementation of the GAPIT software package.

## Introduction

Genome-Wide Association Study (GWAS) has become the leading method to identify genes underlying human diseases and agriculturally important traits. However, the genetic variants identified so far only explain a small portion of phenotypic variation [1]. Rare genes and genes without large effect still remain unidentified due to lack of statistical power [2]. Statistical power is determined by many factors such as gene effect, allele frequency, sample size, marker density, and null distribution of type I error [3]. Inflation of type I error (false positives) leads to more false discoveries than expected [4], [5].

Population stratification and cryptic relationships are two common reasons for the inflation of false positives [6], [7]. Compared to the general linear model (GLM), the Mixed Linear Model (MLM) method effectively eliminates false positives by incorporating these two factors simultaneously [8]. The population stratification is fit as a fixed effect through population structure [6] or principal components [9]. The cryptic relationship among individuals is joined with variance components to collectively define variance and covariance of the random genetic effects from individuals.

The number of individuals in the population largely determines the size of a MLM equation [10]. The computing complexity of solving a MLM is a cubic function of the number of individuals. It is prohibitive to solve a MLM with large number of individuals, especially with iterations to estimate unknown variance components [11]. Several advances have partially solved the computational problem. The Efficient Mixed-Model Association (EMMA) algorithm turns the two-dimensional optimization of genetic and residual variance components into one dimensional optimization by deriving the likelihood as a function of their ratio [12].

Efforts have been made to change the computational function from cubic to quadratic, especially for marker screening, which dominates the entire computation for data with high marker density. The Population Parameter Previously Determined (P3D), or Efficient Mixed-Model Association eXpedited (EMMAX), estimates variance components (or their ratio) only once and then fixes them to test genetic markers [13], [14]. Furthermore, an exact method, Genome-wide Efficient Mixed-Model Association (GEMMA), was developed to estimate the population parameters for each testing marker with the similar computational efficiency of P3D or EMMAX [15].

The method of compressed MLM [13] clusters individuals into groups and fits the groups as the random effect. The computing complexity function is thus reduced from the cubic of the number of individuals to the cubic of a smaller number of groups. However, the cubic property still remains. In practice, the maximum compression (i.e., the average number of individuals per group) observed is only about twenty-fold [16]. Consequently, solving a MLM is still prohibitive with extremely large numbers of individuals.

The Factored Spectrally Transformed Linear Mixed Model (FaST-LMM) partitions the cubic function of computing complexity as the product of two parts: 1) the number of individuals and 2) the square of the rank of the relationship among individuals [17]. When all the genetic markers (usually much larger than the number of individuals) are used to define the relationship among individuals, the kinship among individuals has full rank (i.e., is the same as the number of individuals). The computing complexity is still cubic to the number of individuals. Using a small subset of randomly selected markers to define a rank-reduced relationship has been suggested [17]. When the small subset has a constant number of Single Nucleotide Polymorphisms (SNPs) relative to the number of individuals, the computing complexity becomes linear to the number of individuals. The authors of FaST-LMM show a few examples using a small subset of randomly selected markers to define kinship that have similar results to those using all genetic markers [17]. Further the study demonstrated that a small set of associated genetic markers has better statistical power than a small set of genetic markers selected randomly. The small set of associated genetic markers are used in such way that some of these markers are removed for defining individual relationship if they are from the same region of the testing markers (e.g., within 2 Mb) [18]. The size and content of the set of markers selected becomes critical for computing speed and statistical power.

In this study, we developed a method that dramatically reduces the number of genetic markers used to define individual relationships and remarkably increases statistical power. First, we divide the whole genome into small bins. Each bin is represented by the most significant marker. Second, we select only the influential bins. Third, we use a maximum likelihood method to optimize the size and number of bins selected as the pseudo Quantitative Trait Nucleotides (QTNs) underlying the phenotypes. Fourth, in the final test of each marker, the small set of markers is used to define the relationship among the individuals by excluding the markers that are in Linkage Disequilibrium (LD) to the testing marker, regardless local distance. We call the algorithm the Settlement of MLM Under Progressively Exclusive Relationship (SUPER).

## Materials and Methods

### SUPER method

We developed the SUPER method in the framework of a standard MLM approach, which decomposes the observation (**Y**) into fixed effect (**β**), random genetic effect (**u**) and residual (**e**) as follows.
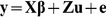
(1)where **u** is a vector of size *n* (number of individuals) for unknown random polygenic effects having a distribution with mean of zero and covariance matrix of 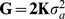, where **K** is the kinship (co-ancestry) matrix with element **K** *_ij_* (i, j = 1, 2, …, n) calculated from genetic markers, and is an unknown additive genetic variance. **X** and **Z** are the incidence matrices for **β** and **u**, respectively, and random residual effects **e** are normally distributed with zero mean and covariance 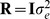, where **I** is the identity matrix and is the unknown residual variance. Solving equation (1) involves determining all the unknown parameters under which the observations (**y**) have the maximum likelihood, defined as the following: 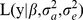
(2)


To perform a GWAS, marker effect (**v**) is added to equation (1), one at a time: 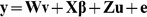
(3)where **W** is the incidence matrix for **v**. Solving equation (3) by using P3D [13] or EMMAX [14] only involves optimization of **v** and **β** to optimize following likelihood: 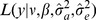
(4)where, 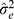 are estimates to maximize equation (2).

Kinship (K) is a known parameter, which is derived from genetic markers. Consequently, different sets of genetic markers create different kinships. This is the only difference among all the methods compared in this study. We used the efficient algorithm [19] of Van Raden et.al. (implemented in GAPIT [20]) to calculate the kinship matrix. The first method is to use the QTNs only. The second method is to use all the SNPs including QTNs. The third method is to use all SNPs except QTNs. The second and third methods are barely different when the number of SNPs is large. The fourth method is similar to the first method in respect of using QTNs. The difference is that a QTN is excluded for deriving the kinship when the testing SNP is the same as the QTN. The kinship is called complementary trait specific kinship. The fifth method is similar to the fourth method except that the QTNs are masked and have to be identified by estimation. Therefore, the method can be used in practice where the true QTNs are unknown. We developed a procedure to find QTN-like SNPs, called pseudo QTNs.

Our procedure consists of three steps. The first two steps perform the inclusion of pseudo QTNs. The last step performs GWAS with exclusion of the pseudo QTNs that are in LD with the tested SNP.

Step 1: To sort SNPs on their p values or effects through a preliminary GWAS or genomic prediction for a specific trait.

Step 2: For each bin (segment) on a chromosome, choose the most influential SNP (e.g., with the lowest P value) as the representative for the bin. Then select *s* most influential bins to build kinship. The size of bins and number of bins chosen are treated as parameters to maximize the restricted maximum likelihood for a trait. The *s* selected SNPs (each represent a bin) are then used as a base of a SNP pool to define individual relationships for the later association test. More precisely, we optimize the following likelihood: 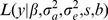
(5)where *s* and *b* are the number and size of bins.

Step 3: When testing a SNP in equation (3), we exclude the SNPs in the SNP pool that are in LD with the testing SNP to derive a complementary trait specific kinship. We call this method as the Settlement Under Progressively Exclusive Relationship (**SUPER**).

Solving equation (3) only involves the optimization of **v** and **β** to optimize following likelihood:
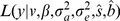
(6)


Where 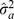, 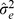 and 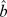 are estimates to maximize equation (5).

### Real Data

Six published datasets from dog, maize, rice, *Arabidopsis*, mouse, and human were examined. The datasets from dog, maize, and rice were the same datasets used in our previous study [13], [16]. The dog dataset was sampled from a dataset used for mapping Quantitative Trait Loci (QTLs) underlying canine hip dysplasia [21] and a dataset used to estimate heritability of canine hip dysplasia [22]. The data contained 292 dogs from two breeds (Labrador Retriever and Greyhound) and their crosses (F_1_, F_2_, and two backcrosses). All dogs were genotyped with 23,500 SNPs at genome-wide coverage.

The maize data contained 282 inbred lines. The genotypes (2,911 SNPs) were released as a tutorial dataset of the TASSEL and GAPIT software packages [23].

The rice data contained 374 inbred lines, 50,000 SNPs randomly sampled from the one million SNPs from genotyping by sequencing technology [16].

The *Arabidopsis* dataset included 199 landraces genotyped by 216,130 SNPs [24]. We randomly sampled 50,000 SNPs for this study.

The mouse data contained 688 34th generation advanced intercross lines (AIL) derived from two inbred strains (SM/J and LG/J). The genotype data contained 3,117 SNPs [25]. The methamphetamine-induced locomotor activity on day 3 was used to compare SUPER with other methods.

The Human Framingham Heart Study (FHS) data were downloaded from the database of Genotypes And Phenotypes (dbGAP) databases (phg000005.v5). The total Cholesterol (Offspring exams 7) was used as the phenotype for the association study. The present study sample comprised 806 FHS offspring participants who were genotyped using the 100K Affymetrix GeneChip and have fasting blood lipid traits for exams 7. We imputed the missing values using mean values by the program GCTA [26]. The genotype data consist of 57,581SNPs on 22 autosomes after exclusion of rare SNPs with Minor Allele Frequency(MAF) less than 0.1 and SNPs with missing genotypes more than 5%. We adjusted the test to control for age, gender, and body mass index to perform GWAS.

### Phenotype simulations

A set of SNPs was randomly sampled as causal QTNs for the simulated traits (27, 20, 24, and 20 QTNs for maize, *Arabidopsis*, rice, and dog, respectively). The location of QTNs were restricted under two scenarios. One scenario was implemented for all the species without any restriction, e.g., a QTN could be any SNP. The other scenario was implemented on the maize dataset only where the last chromosome was excluded to sample QTNs. The last chromosome in the second scenario was used to investigate the effect of a clear null distribution, i.e., no genetic correlation existed between QTNs and non-QTN SNPs.

The distribution of these QTN effects followed a normal distribution with a mean of 0 and variance of 1. Phenotypes were simulated as the following equation: y =  additive + residual. For each individual, the total additive effect is calculated as the sum of additive effects across all QTNs. The residual variance was calculated as V*e* =  V*a*(1-h2)/h2, where V*a* is the additive genetic variance and h2 is the heritability. A residual error following a normal distribution with mean of 0 and variance of Ve was added to the total additive effect to form the simulated phenotype for each individual. Heritability was set to 0.75 for examination of statistical power in all datasets. Another five levels of heritability (h2 = 0, 0.25, 0.4, 0.5 and 1) were set to further compare the statistical power of SUPER with other methods by using the maize dataset.

### Null distribution and power examination

The association tests on the markers were performed by conducting F tests. In the scenario that sampled QTNs without any restriction, the empirical distribution of the non-QTN markers was used as the null distribution of type I error. For the second scenario—last chromosome was excluded for sampling QTNs—the empirical distribution of the markers on the last chromosome was used as the null distribution of type I error. The power is examined as the proportion of QTNs that pass a testing threshold for a given type I error (5%). A total of 100 replications were conducted for each method and the average over the 100 replicates was reported.

### Ethics statement

All the datasets analyzed herein have been previously published. This study did not obtain actual samples from human or animals.

## Results

Through simulations, we demonstrated that the effective components in the small set of selected genetic markers are the QTNs underlying a trait. To remove the confounding between the QTNs and testing markers, the exclusion of QTNs is more effective when LD is used instead of local distance. We examined our proposed method for the practical situations where QTNs are unknown.

**Figure 1:**
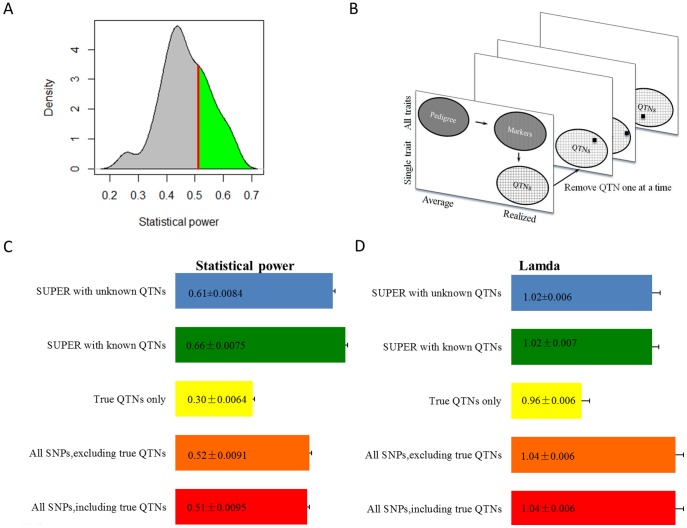
Conception and performances of different methods. **A**) Distribution of statistical power by using a kinship derived from a set of SNPs selected randomly. The dataset contained ∼3,000 SNPs genotyped on 282 maize inbred lines. The number of selected SNPs was the same as the number of individuals used to derive kinship. Power was examined on a trait simulated from 27 causative mutations, i.e. Quantitative Trait Nucleotide (QTNs), sampled from the ∼3,000 SNPs except the ones on the last chromosome. The SNPs on the last chromosome were used to derive the null distribution of Type I error. The heritability of the trait was set to 0.75. A total of 100 replications were conducted. The average and the median power are 0.476 and 0.444. The power of using kinship derived from all SNPs is 0.511 (red line). **B**) Conception of kinship for association study. Pedigree is the first available information used to calculate kinship. It is the expectation for a pair of individuals to be identical by descent at any locus, (e.g., full siblings have a kinship of 50% in cases of no inbreeding). Pedigree kinship can be used across traits. A realized kinship derived from genetic markers covering entire genome is more precise than pedigree based (e.g., full siblings could have a kinship of 60% - or 40% - instead of 50%). However, it is still general and can be used for all traits. A complete trait specific realized kinship is using all the QTNs underlying the trait. This complete trait specific kinship is ideal for genome prediction, but not for GWAS. The ideal kinship for GWAS is its complement (using all QTNs except the one being tested) to remove the confounding between the kinship and the tested SNPs. **C**) and **D**) display the performance of statistical power and effectiveness of genomic control of inflation factor by using different kinship. The statistical power is about 50% when using all the SNPs. Inclusion or exclusion of the 27 QTNs did not have a significant impact. When only the 27 QTNs were used to derive a complete trait specific kinship, the statistical power was dramatically reduced to 30%. When each of the 27 QTNs was tested by using the complementary trait specific kinship derived from the other 26 QTNs (SUPER with known QTNs), the statistical power was boosted to 66%. A statistical power of 61% was retained by using SUPER with masked QTNs. The genomic control of SUPER was similar with known QTNs and with masked QTNs, closer to expectation (1.00) than other methods.

We compared SUPER and other popular mixed model methods through a series of simulations. The difference among these methods is how to build kinship. We showed that a small subset of randomly selected genetic markers will not always produce the equivalent statistical power compared to using all genetic markers (**Figure 1a**). The average statistical power of the small subset of randomly selected genetic markers was significantly less than the power by using all genetic markers (p<0.01). The statistical power was about 50% when using all the markers in a maize dataset with 282 individuals. It does not make difference to include or exclude QTNs as the number of markers is usually much larger than the number of QTNs underlying a trait. Exclusion of all the markers in LD with QTNs, does not make difference compared to using all the markers to build kinship (**Figure 1c** **,** **Table 1**).

**Table 1:** Statistical power of using different kinship for four species (*Arabidopsis*, Rice, Dog and Maize).

Method to build kinship	Arabidopsis	Rice	Dog	Maize
All SNPs, including true QTNs	0.63±0.0070	0.52±0.0063	0.59±0.0079	0.51±0.0095
All SNPs, excluding true QTNs	0.63±0.0072	0.52±0.0061	0.59±0.0083	0.52±0.0091
True QTNs only	0.42±0.0066	0.29±0.0064	0.40±0.0083	0.30±0.0064
SUPER with known QTNs	0.75±0.0065	0.65±0.0057	0.72±0.0076	0.66±0.0075
SUPER with unknown QTNs	0.72±0.0063	0.60±0.0059	0.68±0.0078	0.61±0.0084

A set of SNPs was randomly sampled as causal QTNs for the simulated traits (0.04%, 0.05%, 0.085% and 1%, of the total number of SNPs for *Arabidopsis*, Rice, Dog, and Maize, respectively). The statistical power was estimated with heritability of 0.75. Power is defined as the proportion of QTNs detected under type I error of 5%. A total of 100 replications was conducted for each method. The statistical power shown here is the average of 100 replications.

In the above simulation study, 35% of the time the small set of randomly selected SNPs had higher power than using all SNP kinship. This finding indicates that the gold-standard kinship of using all SNPs is definitely not the best choice. So, the interesting question is: what type of small subset of SNPs produces higher power than using all the SNPs? We were motivated by the fact that a trait specific kinship derived from weighted SNPs has better prediction accuracy than the kinship derived from all the SNPs in genomic prediction [27].

However, when we applied kinship from all the QTNs for GWAS, we found that statistical power decreased to about 30%, which was much lower than using kinship derived from all SNPs. This result is not surprising because the kinship derived from all QTNs is confounded with the effect of the tested SNP if this SNP is one of the QTNs.

This finding confirmed the strategy for selecting the kinship method for GWAS. When testing a SNP, we remove the SNP from the QTN list if the SNP is a QTN. We then use the remaining QTNs to derive a complementary trait specific relationship for the SNP (**Figure 1b**). When the complementary trait specific relationship is applied to GWAS, statistical power is boosted to 66% for the 282 maize dataset, which is much higher than using all SNPs.

For the real situation, where QTNs are unknown, we developed an algorithm to derive a set of pseudo-QTNs for the SUPER method. The algorithm involves three steps. The first step is to perform a preliminary GWAS to sort SNPs. The second step determines the size and number of bins that give the maximum likelihood for a specific trait. Then, for each bin, the most associated SNP is used as the pseudo-QTN to represent that bin. The size and number of bins are the two parameters chosen for optimization. The third step is to perform the complementary process in GWAS by excluding the pseudo-QTNs that are in LD with the tested SNP. The remaining pseudo-QTNs are used to define the complementary relationship among individuals. In the simulation study where the QTNs were masked, we obtained a statistical power of 61%, lower than the situation with known QTNs, but still much higher than using all SNPs (**Figure 1c, 1d**).

**Figure 2:**
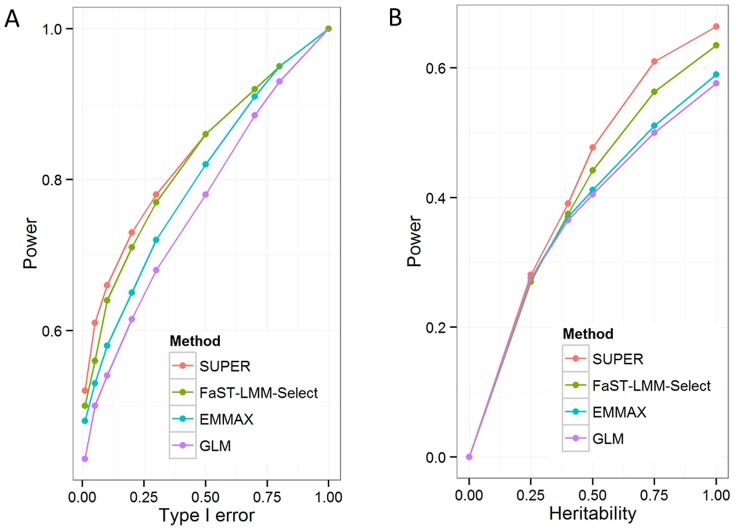
Statistical power under different ranges of type 1 error and heritability. **A**) Statistical power was examined on a trait simulated from 27 causative mutations (QTNs) sampled from SNPs on chromosomes 1 to 9 in maize data. The SNPs on the last chromosome (10) were used to derive null distribution. Power was defined as the proportion of detected QTNs under type I error of 5%. A total of 100 replications was conducted for each method. The heritability of the trait was set to 75%. Four methods were examined: 1) SUPER; 2) EMMAX; 3) FaST-LMM-Select; and 4) General linear model (GLM). **B**) Statistical power of four methods under different heritability levels. The four methods are SUPER, LMM-Selected, EMMAX and GLM.

We extended our examination of statistical power against the type I error for four methods: SUPER, FaST-LMM-Select, EMMAX, and GLM (**Figure 2a**). The SUPER method is consistently better than the others over the entire range of type I errors. The GLM is consistently the worst. The FaST-LMM-Select and EMMAX performed better than GLM. We also compared the statistical power under different levels of heritability. When a trait is more heritable, (e.g., heritability 0.25), the four methods perform differently from each other. FaST-LMM-Select performs better than EMMAX and GLM. SUPER performs better than FaST-LMM-Select (**Figure 2b**).

We explored several ways to reduce the computing time of SUPER. First, we examined the effect using P3D/EMMAX [13], [28]. We found SUPER works well with P3D/EMMAX to reduce computing time and retain similar statistical power. No significant difference (p>0.05) in power was found whether we used P3D/EMMAX or not. Thus, re-estimating population parameters (e.g., genetic variance, residual variance, or their ratio) for testing each SNP is unnecessary. This completely eliminates the iteration time to optimize these population parameters for screening SNPs (**Figure S1**).

Second, we explored speeding up computation by using a fast method to derive the P values at the first stage of SUPER. Three methods were compared: GLM, MLM [8], and Compressed MLM (CMLM) [13]. The GLM method is much faster than the other two methods. Although using GLM in the first step tends to have less power than using the other two methods, the difference is not significant. Thus, even when using GLM to keep computing cost low, the statistical power of the SUPER method is not affected significantly (**Figure S2**).

Third, we provided a procedure to determine the threshold of LD between tested SNPs and QTNs. When the threshold is too high (e.g., r^2^ = 100%), QTNs are barely removed. The result should be similar to the complete trait specific kinship. In the opposite case, where the threshold is too low (e.g., r^2^ = 0.01%), QTNs are hardly survived in the exclusion process. The kinship matrix does not retain much information and the results would be similar to the GLM. We observed that a threshold of r^2^ = 10% was best for both maize and rice. This threshold also worked well for the other species (dog and *Arabidopsis*) we examined (**Figure S3**). Nevertheless, this finding only provides guidance for the optimizations, which might be necessary for other populations or species.

We examined our findings for a variety of circumstances. We verified the effect of the correlation between QTNs and the non-QTN SNPs. The non-QTN SNPs were used to derive the empirical null distribution of type I error. Two scenarios were examined. In the first scenario, no correlation was found because QTNs and non-QTN SNPs were sampled from different chromosomes. In the second scenario, correlation was possible because random sampling might place QTNs and non-QTN SNPs next to each other. We observed that, in either case, our findings still held. That is, 1) SUPER with known QTNs had the highest statistical power, 2) complete trait specific kinship had the lowest power, 3) kinship from all SNPs was in the middle, and 4) SUPER with unknown QTNs fell between SUPER with known QTNs and the kinship from all SNPs (**Figure S4**).

We then examined the impact of the magnitude of QTN effect (**Figure S5**) and heritability (**Figure S6**). We observed the same trend in statistical power as above. SUPER with known QTNs is the best and SUPER with unknown QTNs is the second best.

**Figure 3:**
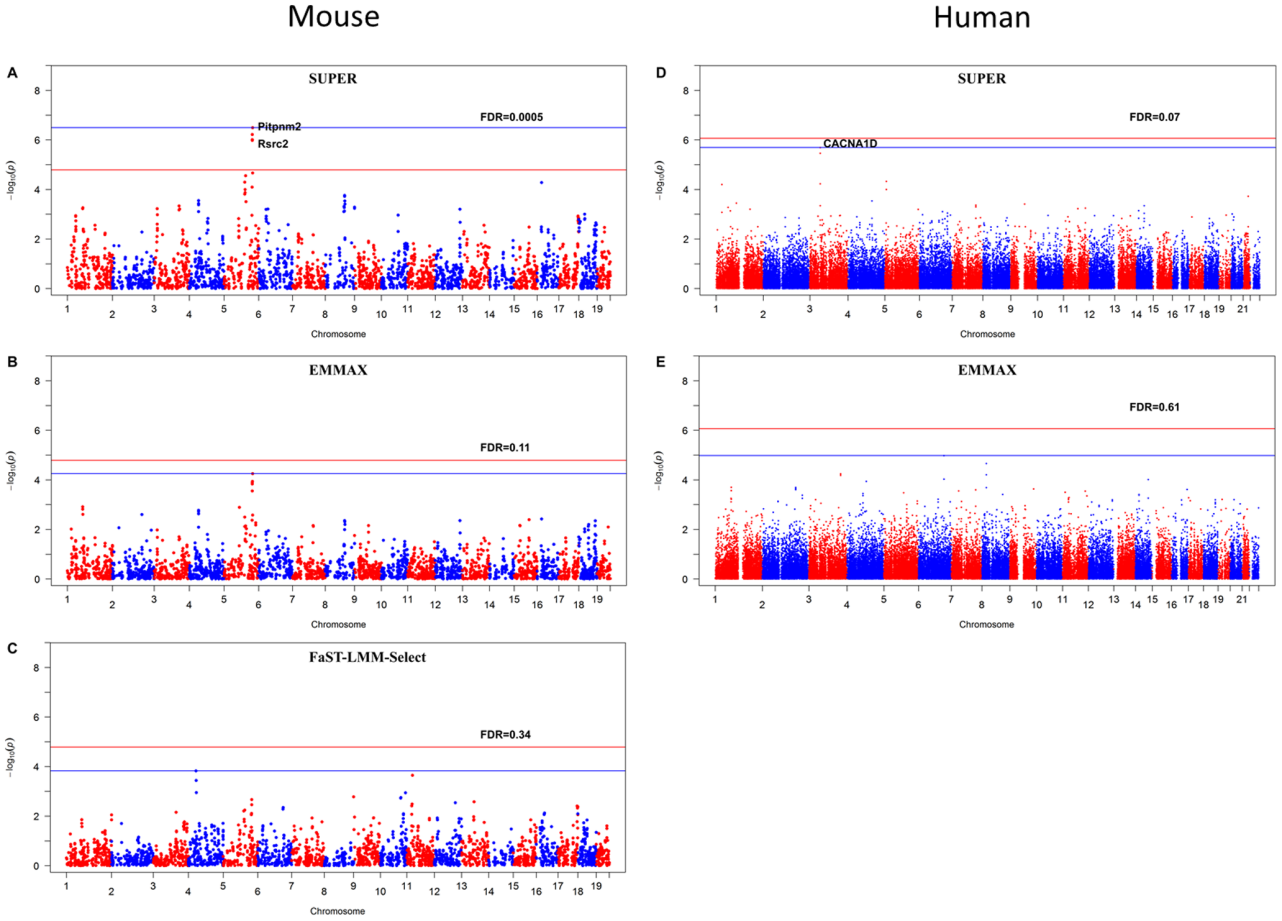
Results of association studies on real mouse and human phenotypes. The mouse phenotype is methamphetamine-induced locomotor activity on day 3 measured on 688 Advanced Intercross Lines (AIL). The human phenotype is cholesterol collected by the Framingham Heart Study (FHS) Project. Each dataset was analyzed with three different methods (SUPER, EMMAX, and FaST-LMM-Select) except the combination between FaST-LMM-Select and human data. The missing genotypes in the human data were imputed in format of dosage, which is not accepted by FaST-LMM-Select. The most significant SNP is highlighted by a horizontal blue line and labeled by its corresponding False Discover Rate (FDR). The p value threshold of 0.05 (after bonferroni multiple test correction) is indicated by a horizontal red line.

We expanded the comparisons of SUPER with EMMAX and FaST-LMM-Select to real traits. The first is from the Advanced Intercross Line (AIL) mouse data [25]. Manhattan plots of all mouse data for the three different methods are shown in **Figure 3** (A to C). The SNPs identified using SUPER at a Bonferroni correction threshold of 0.05 and a False Discovery Rate (FDR) less than 0.1 are listed in **Table 2**. Using the SUPER method, we identified all the associations previously detected by the original paper. Two of these significant SNPs were located in known genes (Rsrc2 and Pitpnm2) [29], [30]. EMMAX and FaST-LMM-Select did not identify significant SNPs that reached the same threshold.

**Table 2:** SNPs found to be significant by SUPER and other three methods for AIL mouse data.

SNP	Chromosome	Position	EMMAX	Fast_LMM_Select	SUPER	Gene
5-122651666	5	125405148	5.60E-05	2.15E-03	**3.22E-07**	
mUC-rs13478501	5	124051672	1.47E-04	2.81E-02	**6.09E-07**	
NES14715162	5	124119050	1.28E-04	8.81E-03	**9.82E-07**	*Pitpnm2*
5-122053167	5	124768242	1.15E-04	7.75E-03	**9.98E-07**	
5-121026072	5	123740172	2.80E-04	4.46E-02	**1.04E-06**	*Rsrc2*

P values that reached the Bonferroni correction threshold (1.6E-5) are shown in bold.

The second real data is from the Human Framingham Heart Study (FHS) project. Missing genotypes were imputed. As FaST-LMM-Select does not accept dosage genotypes, the comparison was performed between SUPER and EMMAx. Manhattan plots of total cholesterol for SUPER and EMMAX are shown in **Figure** 3 (D and E). Neither method identified significant SNPs that reached the Bonferroni correction threshold of 0.05. However, using the SUPER method, we identified two significant SNPs (rs1599231 and rs898408) at a FDR less than 0.1. The identified SNPs are located in known gene (*CACNA1D*) associated with cholesterol [31]. EMMAX did not identify significant SNPs at this FDR threshold.

With the SUPER method, the restriction of the computationally efficient FaST-LMM method is no longer a problem. Their joint usage retains the similar computing speed while remarkably improving the statistical power.

## Discussion

The concept of complementary trait specific kinship reflects a landmark in the development of kinship. As essential information in population and quantitative genetics, kinship is traditionally derived from pedigree as an expected chance that two individuals share the same allele by descent [32]. The pedigree-kinship relationship has been widely used to study human diseases and predict breeding values for animals and plants [33], [34].

An alternative way to derive kinship is to rely on genetic markers [35], [36]. This marker-based kinship more precisely specifies the actual difference between individuals. Some of these differences are not distinguishable using the kinship derived from pedigree [37]. For example, all full siblings have the same relationship with each other based on pedigree. These relationships become distinguishable with genetic markers. The realized kinship revealed by markers could be quite different from the kinship derived from pedigree due to factors like allele sampling and segregation distortion [37]. The realized kinship is superior to the pedigree kinship for ranking individuals for their genetic merit [38]. When the realized kinship is used jointly with population structure in a MLM for GWAS, it performs well in controlling false positives [8]. Furthermore, the realized kinship can be derived for a specific trait using the markers that are influential to the trait. This trait specific kinship produces higher prediction accuracy than the universal realized kinship [27].

Obviously, the best kinship to define individual genetic relationship on a complex trait is the one derived from all the QTNs underlying the trait as they define it [39]. Adding additional SNPs (non QTNs) would dilute the actual relationship. Complete trait specific kinship works the best for genomic prediction [27]. But, when used for GWAS, the markers defining the kinship are confounded with the tested markers, consequently decreasing the statistical power of GWAS.

However, less obvious, is that a small proportion of randomly sampled SNPs would have higher statistical power than using all SNPs. The increased power might result from the combination of the following factors: 1) sampled SNPs contain QTNs or SNPs in LD with QTNs, 2) fewer non-QTN SNPs result in less dilution, and 3) a portion of QTNs, or SNPs in LD with QTNs are excluded and become more detectable.

There is a random chance that a small subset of SNPs selected randomly could have higher power than using all SNPs. In general, the randomly selected subsets of SNPs have less power. Therefore, randomly selecting a small set of SNPs is unsafe. The goal of this study was to find a better method to find subsets. Ideally, the subset contains fewer SNPs than number of individuals and has the same or higher power than using all the SNPs.

FaST-LMM-Select has been undertaken to find small subsets of SNPs [18]. Similar to SUPER, the strategy works best for a scenario in which a complex trait is controlled by genes with large effect, small effect, and anything between. For an extreme case having only a few (e.g., 1 to 3) genes with major effects and the rest (e.g., 500) with very small effects, the power will be saturated to 100% for the major genes even with a small sample and a simple method. However, the rest of the genes will have no power regardless of method, including FaST-LMM-Select or SUPER proposed in this study if the sample is not large enough.

Our study was unique in a several ways. Overall, our study gives the biological, inside-view for the statistical phenomena observed in the FaST-LMM-Select study. Through a series of simulations, we proved that their finding — that using a small set of randomly selected SNPs generates the equivalent statistical power as using all the SNPs — is not always true. In fact, statistical power can be reduced significantly. This result is not surprising as a small random sample of SNPs is less informative than using all the SNPs [40].

Furthermore, we explained why the kinship for GWAS should be specific for a trait and complementary to a testing SNP. We started with known QTNs and showed how different scenarios impact statistical power, such as using all QTNs or using QTNs excluding the one being tested. These studies demonstrate how the inclusion of all QTNs confounds with the effects of testing SNPs when compared to all SNPs and how the exclusion of QTNs eliminates the confounding.

We applied the method derived from situations with known QTNs to real-life situations with unknown QTNs. We developed the algorithm to find their representatives (pseudo-QTNs) and demonstrated that the SUPER approach has statistical power close to that achieved with known QTNs. We determined the set of pseudo-QTNs by optimizing bin size and bin number to define the trait through a method of maximum likelihood. This set of pseudo-QTNs is the best combination among all SNPs compared with the FaST-LMM-Select study, which selects only the top significant SNPs. That we demonstrated a higher power by using SUPER, compared to FaST-LMM-Select, is not surprising.

The top significant SNPs selected in the FaST-LMM-Select study likely include multiple SNPs from each association peak in GWAS. These SNPs are in strong LD among themselves. One obvious disadvantage is that this SNP selection method causes severe dilution. The other disadvantage is that computational time increases by including more SNPs than necessary. The SUPER method avoids this problem by using the pseudo-QTNs. Only one SNP is selected from many SNPs on each peak. Consequently, the optimum number of SNPs used to derive kinship is much smaller.

Moreover, LD is not only caused by local genetic linkage. Many other factors can cause LD between SNPs (e.g., population structure), even when SNPS are on different chromosomes. Therefore, our complementary process is performed genome-wide, and is not limited to the nearby SNPs (FaST-LMM-Select uses a 2 cM interval).

FaST-LMM-Select uses an arbitrary interval (2 cM) as the threshold of exclusion for LD. We use a precise LD parameter (R^2^). We demonstrated that R^2^ of 10% was robust enough to give the highest statistical power in all species we examined.

Last, but certainly not least, FaST-LMM-Select complements our method. FaST-LMM-Select provides an elegant algorithm to reduce computation time by conducting single value decomposition only once. Thus, the joint usage of these two methods will provide powerful and flexible tools.

We anticipate that the SUPER method could be used jointly with the CMLM to further improve statistical power. Each individual would still have its group assignment. However, the kinship of groups would be replaced by the assignment of individual QTN to groups. The effects of different assignments remain an open research question.

SUPER has been implemented in the publicly available software package, GAPIT. This method makes it possible to detect a gene with smaller samples, or alternatively, to detect a smaller effect gene with the same sample size.

**URLs**: Computer programs (R source code) are available at http://www.zzlab.net/GAPIT/.

## Supporting Information

Figure S1 **P3D (Population Parameter Previously Determined) can be used in SUPER.** Similar to kinship derived from other methods, the statistical power of SUPER with unknown QTNs was the same for using or not using P3D. The other methods include the kinship derived from all the SNPs including true QTNs, the kinship derived from all SNPs excluding true QTNs, SUPER with known QTNs, and the complete trait specific kinship (True QTNs only).
(TIF)

Figure S2 **Effect of the methods to derive the P values at the first stage of SUPER.** Three methods were compared: General Linear Model (GLM), Mixed Linear Model (MLM) and Compressed Mixed Linear Model (CMLM).
(TIF)

Figure S3 **The effect of linkage disequilibrium threshold to exclude QTNs for testing SNPs.** The scenarios were implemented on the Maize, *Arabidopsis*, Rice, and Dog datasets, respectively. When the threshold is large, e.g. r^2^ = 100%, QTNs are barely removed. The result should be similar to the complete trait specific kinship. In the opposite case, when the threshold is too small, e.g. r^2^ = 0.01%, QTNs hardly survived the exclusion process. The kinship does not retain much information and the result would be similar to GLM. Interestingly, we observed that the threshold of r^2^ = 10% work well for all species.
(TIF)

Figure S4 **Effect from the relation between the QTNs and the other SNPs to derive the null distribution of test statistics.** The power was examined on a trait simulated from 27 causative mutations (QTNs) sampled from the Maize dataset under a type I error of 0.05. A total of 100 replications were conducted for each method. No linkage was found between QTNs and the null SNPs in the ideal situation, when the QTNs and the null SNPs were sampled from different chromosomes. In the opposite situation (regular SNPs), when QTNs and the null SNPs were randomly sampled from the entire SNPs, a potential linkage was found between QTNs and the null SNPs. The statistical power was the same between these two scenarios for all methods. These methods include SUPER with known QTNs, SUPER with unknown QTNs, the complete trait specific kinship (true QTNs only), kinship from all SNPs including true QTNs, and kinship from all SNPs except QTNs.
(TIF)

Figure S5 **Statistical power of five methods under different magnitudes of QTN effect.** The power was examined on a trait underlying causative mutations (QTNs) sampled from ∼3000 SNPs in maize. A total of 100 replications was conducted for each method. The statistical power shown here is the average of 100 replications. The heritability of the trait was 50%. The five methods are: 1) complete trait specific kinship (true QTNs only), 2) complementary trait specific kinship with known QTNs (SUPER with known QTNs), 3) complementary trait specific kinship with unknown QTNs (SUPER with unknown QTNs), 4) all SNPs including QTNs, and 5) all SNPs except QTNs.
(TIF)

Figure S6 **Statistical power of five methods under different heritability levels.** The power was examined on a trait simulated from 27 causative mutations (QTNs) sampled from ∼3000 SNPs in maize. A total of 100 replications was conducted for each method. The statistical power shown here is the average of 100 replications. The heritability of the trait varied from 0 to 1. The differences between complete trait specific kinship (true QTNs only) and complementary trait specific kinship (SUPER with known QTNs) were greater when heritability was between 0 and 1. The difference between SUPER with known QTNs and kinship derived from all SNPs increases with heritability. No significant difference was found between kinship derived from all SNPs and all SNPs except QTNs.
(TIF)

## References

[1] Yang J, Benyamin B, McEvoy BP, Gordon S, Henders AK, et al (2010) Common SNPs explain a large proportion of the heritability for human height. Nat Genet 42: 565–569. 20562875 10.1038/ng.608 PMC3232052

[2] Buckler ES, Holland JB, Bradbury PJ, Acharya CB, Brown PJ, et al (2009) The genetic architecture of maize flowering time. Science 325: 714–718. 19661422 10.1126/science.1174276

[3] Pe'er I, de Bakker PI, Maller J, Yelensky R, Altshuler D, et al (2006) Evaluating and improving power in whole-genome association studies using fixed marker sets. Nat Genet 38: 663–667. 16715096 10.1038/ng1816

[4] Moonesinghe R, Khoury MJ, Janssens AC (2007) Most published research findings are false-but a little replication goes a long way. PLoS Med 4: e28. 17326704 10.1371/journal.pmed.0040028 PMC1808082

[5] Ioannidis JPA (2005) Why most published research findings are false. Plos Medicine 2: 696–701. 10.1371/journal.pmed.0020124 PMC1182327 16060722

[6] Pritchard JK, Stephens M, Rosenberg NA, Donnelly P (2000) Association mapping in structured populations. American Journal of Human Genetics 67: 170–181. 10827107 10.1086/302959 PMC1287075

[7] Zhang Z, Buckler ES, Casstevens TM, Bradbury PJ (2009) Software engineering the mixed model for genome-wide association studies on large samples. Brief Bioinform 10: 664–675. 19933212 10.1093/bib/bbp050

[8] Yu JM, Pressoir G, Briggs WH, Bi IV, Yamasaki M, et al (2006) A unified mixed-model method for association mapping that accounts for multiple levels of relatedness. Nature Genetics 38: 203–208. 16380716 10.1038/ng1702

[9] Zhao K, Aranzana MJ, Kim S, Lister C, Shindo C, et al (2007) An Arabidopsis example of association mapping in structured samples. PLoS Genet 3: e4. 17238287 10.1371/journal.pgen.0030004 PMC1779303

[10] Henderson CR (1984) Applications of Linear Models in Animal Breeding. University of Guelph, Guelph, Ontario, Canada.

[11] Gilmour AR, Thompson R, Cullis BR (1995) Average Information REML: An Efficient Algorithm for Variance Parameter Estimation in Linear Mixed ModelsAverage Information REML: An Efficient Algorithm for Variance Parameter Estimation in Linear Mixed Models Biometrics. 51: 1440–1450.

[12] Kang HM, Zaitlen NA, Wade CM, Kirby A, Heckerman D, et al (2008) Efficient Control of Population Structure in Model Organism Association Mapping. Genetics 178: 1709–1723. 18385116 10.1534/genetics.107.080101 PMC2278096

[13] Zhang Z, Ersoz E, Lai CQ, Todhunter RJ, Tiwari HK, et al (2010) Mixed linear model approach adapted for genome-wide association studies. Nat Genet 42: 355–360. 20208535 10.1038/ng.546 PMC2931336

[14] Kang HM, Sul JH, Service SK, Zaitlen NA, Kong SY, et al (2010) Variance component model to account for sample structure in genome-wide association studies. Nat Genet 42: 348–354. 20208533 10.1038/ng.548 PMC3092069

[15] Zhou X, Stephens M (2012) Genome-wide efficient mixed-model analysis for association studies. Nat Genet 44: 821–824. 22706312 10.1038/ng.2310 PMC3386377

[16] Huang X, Wei X, Sang T, Zhao Q, Feng Q, et al (2010) Genome-wide association studies of 14 agronomic traits in rice landraces. Nat Genet 42: 961–967. 20972439 10.1038/ng.695

[17] Lippert C, Listgarten J, Liu Y, Kadie CM, Davidson RI, et al (2011) FaST linear mixed models for genome-wide association studies. Nat Methods 8: 833–835. 21892150 10.1038/nmeth.1681

[18] Listgarten J, Lippert C, Kadie CM, Davidson RI, Eskin E, et al (2012) Improved linear mixed models for genome-wide association studies. Nat Methods 9: 525–526. 22669648 10.1038/nmeth.2037 PMC3597090

[19] VanRaden PM (2008) Efficient methods to compute genomic predictions. J Dairy Sci 91: 4414–4423. 18946147 10.3168/jds.2007-0980

[20] Lipka AE, Tian F, Wang Q, Peiffer J, Li M, et al (2012) GAPIT: genome association and prediction integrated tool. Bioinformatics 28: 2397–2399. 22796960 10.1093/bioinformatics/bts444

[21] Zhou Z, Sheng X, Zhang Z, Zhao K, Zhu L, et al (2010) Differential Genetic Regulation of Canine Hip Dysplasia and Osteoarthritis. PLoS ONE 5: e13219. 20949002 10.1371/journal.pone.0013219 PMC2952589

[22] Zhang Z, Zhu L, Sandler J, Friedenberg SS, Egelhoff J, et al (2009) Estimation of heritabilities, genetic correlations, and breeding values of four traits that collectively define hip dysplasia in dogs. American Journal of Veterinary Research 70: 483–492. 19335104 10.2460/ajvr.70.4.483

[23] Bradbury PJ, Zhang Z, Kroon DE, Casstevens TM, Ramdoss Y, et al (2007) TASSEL: software for association mapping of complex traits in diverse samples. Bioinformatics 23: 2633–2635. 17586829 10.1093/bioinformatics/btm308

[24] Atwell S, Huang YS, Vilhjalmsson BJ, Willems G, Horton M, et al (2010) Genome-wide association study of 107 phenotypes in Arabidopsis thaliana inbred lines. Nature 465: 627–631. 20336072 10.1038/nature08800 PMC3023908

[25] Cheng R, Lim JE, Samocha KE, Sokoloff G, Abney M, et al (2010) Genome-wide association studies and the problem of relatedness among advanced intercross lines and other highly recombinant populations. Genetics 185: 1033–1044. 20439773 10.1534/genetics.110.116863 PMC2907190

[26] Yang J, Lee SH, Goddard ME, Visscher PM (2011) GCTA: a tool for genome-wide complex trait analysis. Am J Hum Genet 88: 76–82. 21167468 10.1016/j.ajhg.2010.11.011 PMC3014363

[27] Zhang Z, Liu JF, Ding XD, Bijma P, de Koning DJ, et al. (2010) Best Linear Unbiased Prediction of Genomic Breeding Values Using a Trait-Specific Marker-Derived Relationship Matrix. PLoS ONE 5. 10.1371/journal.pone.0012648 PMC2936569 20844593

[28] Kang HM, Sul JH, Service SK, Zaitlen NA, Kong S-y, et al (2010) Variance component model to account for sample structure in genome-wide association studies. Nature genetics 42: 348–354. 20208533 10.1038/ng.548 PMC3092069

[29] Kawai J, Shinagawa A, Shibata K, Yoshino M, Itoh M, et al (2001) Functional annotation of a full-length mouse cDNA collection. Nature 409: 685–690. 11217851 10.1038/35055500

[30] Diez-Roux G, Banfi S, Sultan M, Geffers L, Anand S, et al (2011) A high-resolution anatomical atlas of the transcriptome in the mouse embryo. PLoS Biol 9: e1000582. 21267068 10.1371/journal.pbio.1000582 PMC3022534

[31] Kathiresan S, Manning AK, Demissie S, D'Agostino RB, Surti A, et al (2007) A genome-wide association study for blood lipid phenotypes in the Framingham Heart Study. BMC Med Genet 8 Suppl 1S17. 17903299 10.1186/1471-2350-8-S1-S17 PMC1995614

[32] Wright SI (1922) Coefficient of inbreeding and relationship. The American Naturalist 56: 330–338.

[33] Henderson CR (1953) Estimation of Variance and Covariance Components. Biometrics 9: 226–252.

[34] Bernardo R (2003) Parental selection, number of breeding populations, and size of each population in inbred development. Theor Appl Genet 107: 1252–1256. 12928779 10.1007/s00122-003-1375-0

[35] Hardy OJ, Vekemans X (2002) spagedi: a versatile computer program to analyse spatial genetic structure at the individual or population levels. Molecular Ecology Notes 2: 618–620.

[36] Zhang Z, Todhunter RJ, Buckler ES, Van Vleck LD (2007) Technical note: Use of marker-based relationships with multiple-trait derivative-free restricted maximal likelihood. J Anim Sci 85: 881–885. 17085728 10.2527/jas.2006-656

[37] Myles S, Peiffer J, Brown PJ, Ersoz ES, Zhang Z, et al (2009) Association Mapping: Critical Considerations Shift from Genotyping to Experimental Design. Plant Cell 21: 2194–2202. 19654263 10.1105/tpc.109.068437 PMC2751942

[38] Hayes BJ, Visscher PM, Goddard ME (2009) Increased accuracy of artificial selection by using the realized relationship matrix. Genet Res 91: 47–60. 10.1017/S0016672308009981 19220931

[39] Pirinen M, Donnelly P, Spencer CC (2012) Including known covariates can reduce power to detect genetic effects in case-control studies. Nat Genet 44: 848–851. 22820511 10.1038/ng.2346

[40] Yu J, Zhang Z, Zhu C, Tabanao DA, Pressoir G, et al (2009) Simulation Appraisal of the Adequacy of Number of Background Markers for Relationship Estimation in Association Mapping. Plant Genome 2: 63–77.

